# Supplementary material for: The interactome of intact mitochondria by cross-linking mass spectrometry provides evidence for coexisting respiratory supercomplexes
Source: Mol Cell Proteomics. 2017 Dec 8;17(2):216–32. doi: 10.1074/mcp.RA117.000470 (PMC5795388; doi:10.1074/mcp.RA117.000470)
Supplement: Supplemental Data [file supp_RA117.000470_133922_0_supp_23978_fzffwf.zip › spectra_annotation/mito_spectra_annotation/123-1-4-1-4-1.pdf]

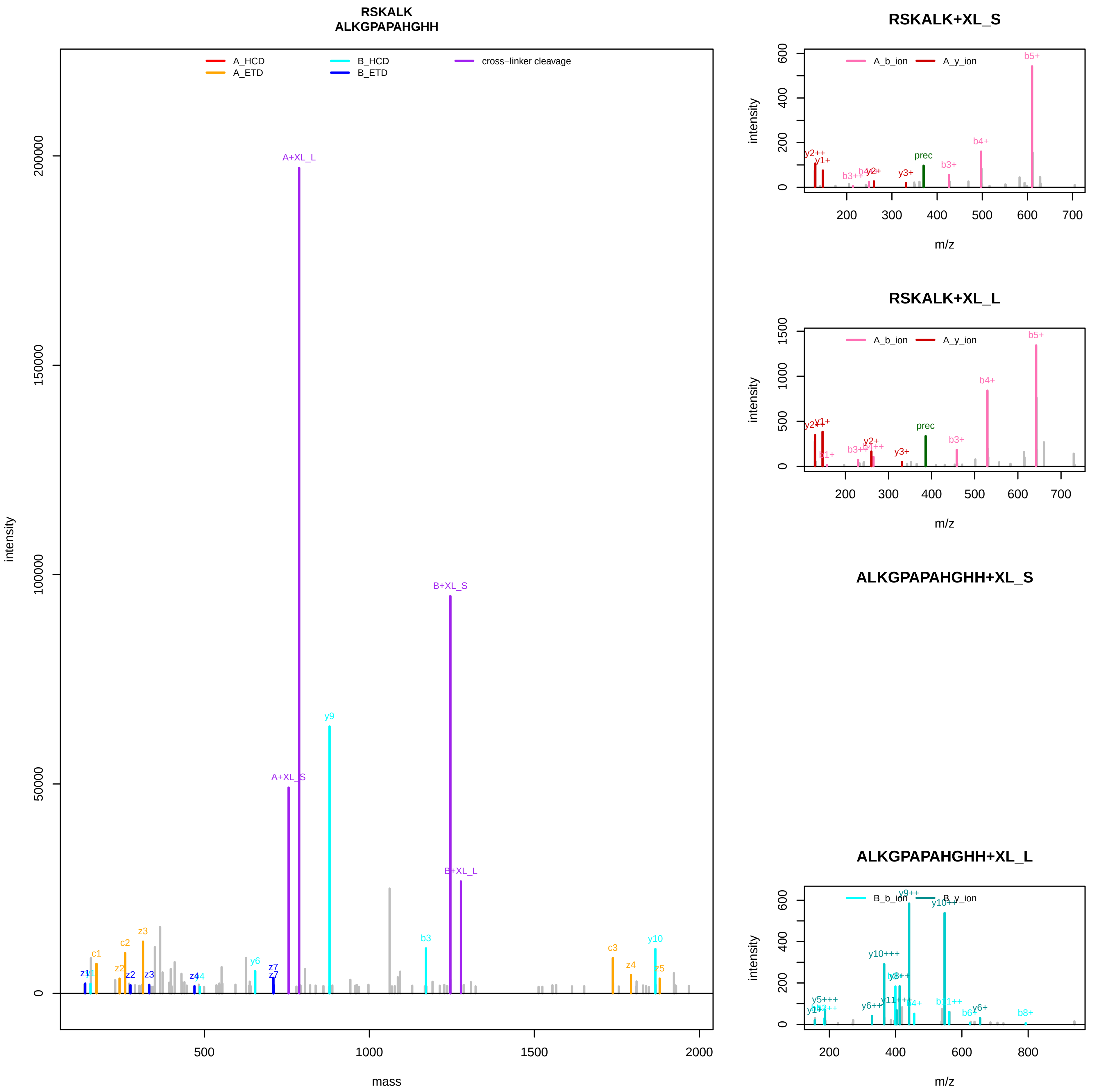

RSKALK
ALKGPAPAHGHH
A_HCD
A_ETD
B_HCD
B_ETD
cross−linker cleavage
A+XL_L
B+XL_S
A+XL_S
B+XL_L
y9
b3
y10
c3
z4
z5
c1
c2
z3
z2
z7
y6
intensity
mass
RSKALK+XL_S
RSKALK+XL_L
ALKGPAPAHGHH+XL_S
ALKGPAPAHGHH+XL_L
A_b_ion
A_y_ion
B_b_ion
B_y_ion
prec
b5+
b4+
b3+
y2++
y1+
y2+
y3+
b1+
y9++
y10++
y10+++
y11+++
b11++
y6+
b8+
y6++
y5+++
m/z
